# VBM with viscous fluid registration of gray matter segments in SPM

**DOI:** 10.3389/fnagi.2013.00030

**Published:** 2013-07-15

**Authors:** Joao M. S. Pereira, Julio Acosta-Cabronero, George Pengas, Li Xiong, Peter J. Nestor, Guy B. Williams

**Affiliations:** ^1^Laboratory of Biostatistics and Medical Informatics, IBILI - Faculty of Medicine, University of CoimbraCoimbra, Portugal; ^2^Department of Clinical Neurosciences, School of Clinical Medicine, University of CambridgeCambridge, UK; ^3^Neurology Department, Zhongnan Hospital of Wuhan UniversityWuhan, China; ^4^German Center for Neurodegenerative Diseases (DZNE)Magdeburg, Germany

**Keywords:** MRI, VBM, SPM, DARTEL, registration, dementia

## Abstract

Improved registration of gray matter segments in SPM has been achieved with the DARTEL algorithm. Previous work from our group suggested, however, that such improvements may not translate to studies of clinical groups. To address the registration issue in atrophic brains, this paper relaxed the condition of diffeomorphism, central to DARTEL, and made use of a viscous fluid registration model with limited regularization constraints to register the modulated gray matter probability maps to an intra-population template. Quantitative analysis of the registration results after the additional viscous fluid step showed no worsening of co-localization of fiducials compared to DARTEL or unified segmentation methods, and the resulting voxel based morphometry (VBM) analyses were able to better identify atrophic regions and to produce results with fewer apparent false positives. DARTEL showed great sensitivity to atrophy, but the resulting VBM maps presented broad, amorphous regions of significance that are hard to interpret. We propose that the condition of diffeomorphism is not necessary for basic VBM studies in atrophic populations, but also that it has disadvantages that must be taken into consideration before a study. The presented viscous fluid registration method is proposed for VBM studies to enhance sensitivity and localizing power.

## Introduction

Imaging biomarkers are of considerable interest in dementia research. Aside from the qualities of the biomarker itself, the method with which it is analysed is crucial to consider as insensitive or unstable methods could mean that real information is lost (false negatives) or that spurious changes (false positives) are reported. Voxel-based image analysis has become a cornerstone of assessing structural imaging biomarkers, yet such methods are typically validated in simulations and the healthy population. The DARTEL algorithm (Diffeomorphic Anatomical Registration Through Exponentiated Lie Algebra) has been presented as an improvement for the registration of gray matter probability maps used in voxel based morphometry (VBM) (Ashburner, 2007) in the software package SPM (http://www.fil.ion.ucl.ac.uk/spm/). One of the key advantages of DARTEL is its explicit search for an inverse consistent, diffeomorphic transformation. This leads to very smooth, large deformation fields that follow elegant mathematical descriptors and that can be easily inverted. Although such characteristics can be useful for applications where it is important to consistently use reverse deformation fields, that is not the case with standard VBM studies of dementia, where most often scans are simply registered to standard space and nothing more is done with the deformation fields (aside from their use for modulation for volume change) (Ridgway et al., 2008). Moreover, a previously published study (Pereira et al., 2010) demonstrated variability in the registration accuracy both by region and disease categorization, raising questions about the spatially variant sensitivity of the resulting VBM significance maps. Even though DARTEL shows improved performance when using preprocessed scans (bias corrected and skull stripped), the associated VBM results showed apparent false positives when compared to the standard SPM5 results using the same scans (Pereira et al., 2010). The motivation for the present study was to explore a simpler, standard registration algorithm without diffeomorphism, in order to assess whether DARTEL's stringent mathematical approach, is in effect inappropriately regularized for use in neurodegenerative disease research. The approach taken in the present study makes use of a long established high degrees of freedom registration algorithm as an alternative: a viscous fluid registration (Christensen et al., 1996). The novelty in the current study compared to past fluid registration studies, however, is its use as an additional registration step, on top of the standard registration in SPM. The hypothesis is that by relaxing the severe regularization constraints intrinsic to DARTEL, the viscous fluid algorithm will be able to account for more anatomical variability, while also preserving structural detail post-registration. This is especially applicable to atrophic brains, which may require both finer (more local) and more extreme deformations than the ones permitted by DARTEL. The resulting VBMs will be more sensitive and more anatomically meaningful than either VBM with DARTEL or standard VBM. As the viscous fluid methods are able to account for finer deformations than the limited degrees of freedom discrete cosine transform (DCT) used in unified segmentation (Ashburner and Friston, 2005), this study considered the utility of applying the former as an additional step to include after SPM's registration. This was implemented as a hierarchical registration model in which a low degrees of freedom algorithm (DCT in unified segmentation) is applied first in order to account for gross differences between the target and the subject, and then a high degree of freedom method (viscous fluid) is applied to address the finer details. The concept of working on top of already registered gray matter probability maps is justified by the fact that DCT registration is unable to account for all anatomical differences between the subject and the template (Ashburner and Friston, 1999). In short, rather than using a complicated, mathematically rich approach like DARTEL in order to achieve smooth deformation fields that might be of little benefit to a real clinical study, better results might be achieved by adding an extra step after the unified segmentation.

The method presented in this study was tested using real datasets from neurodegenerative studies because these are precisely the datasets where such algorithms find application as VBM studies. This is of paramount importance because other registration approaches, notably DARTEL, have been validated in abstract mathematical frameworks or on healthy volunteer data (Ashburner, 2007; Yassa and Stark, 2009) and then accepted as clinical tools without thorough testing in clinical settings. The clinical plausibility of the resulting statistical maps of VBM, as compared with known patterns of atrophy from other assessments, is a significant consideration if these methods are to be robust for clinical studies. In the current study, the results were assessed in comparison to prior knowledge of disease atrophy patterns and manual hippocampal volumetry in patient groups. Finally, fiducial markers were placed in a subset of subjects so as to directly compare the method to the standard unified segmentation and DARTEL methods.

## Materials and methods

All algorithms presented in this section were written in Matlab7, and run on a Dual Xeon 3.2 GHz Intel X86 64 bit processor with 4GB RAM running GNU Debian Linux version 3.1, except where otherwise stated. The code of the fluid registration algorithm presented in this paper can be found in http://www.uc.pt/en/fmuc/ibili/Archives/Articles/JPereira/MiMe.

### Viscous fluid algorithm

A viscous fluid algorithm was implemented in order to register the gray matter probability maps generated by SPM8 (http://www.fil.ion.ucl.ac.uk/spm/software/spm8/). Viscous fluid registration assumes a subject's brain *S* to behave as a viscous fluid when being registered to a target space *T*, with each point of the subject scan being deformed by the action of an external driving force field **F**. This force field is cancelled out at equilibrium by the internal forces of the fluid body, as described by the Navier-Stokes equation:

(1) $$\mu \nabla^{2}v(x,\;t)+(\mu +\lambda )\nabla (\nabla \;\cdot \;v(x,\;t))+F(x,\;u(x,\;t))=0$$

with:

(2) $$v(x,\;t)=\frac{du(x,\;t)}{dt}$$

In Equation 1, **F**(**x**, **u**(**x**, *t*)) is the external force acting on the body deformed by the displacement field **u**(**x**, *t*) at location **x** at time *t*, ∇ is the gradient operator, ∇^2^ is the Laplacian operator, and μ and λ are the viscosity constants. From this point on, dependencies will be dropped for the sake of simplicity [e.g., **v**(**x**, *t*) will be written as **v**]. For the external forces calculation, a simple difference metric was used, based on the difference between the target and the deformed subject, multiplied by the gradient of the latter (Christensen et al., 1996).

As a linear differential equation, and using finite differences to discretize it (Press et al., 1992), Equation 1 can be written as:

(3) Lv=F

where **L** is a linear operator, **v** is the velocity field and **F** is the external force field. This system can be either explicitly solved or a solution can be estimated using approximations to the linear operator **L**.

The explicit solution requires the use of the successive over-relaxation (SOR) method (Press et al., 1992; Wollny and Kruggel, 2002), which may be too time consuming. One possibility is to speed it up by using adaptive updates (SOR with adaptative updates, SORA) (Wollny and Kruggel, 2002). Another option is to approximate the linear operator **L** with an adequate convolution kernel **K**. As a consequence, the velocity response to each individual force vector can be estimated by its convolution with **K**. It is known that **K** can be a Gaussian filter (Thirion, 1998). This is a simplistic solution that has been shown to cause a decrease in registration quality (Gramkow and Bro-Nielsen, 1997). Another approximation, based on the eigenfunctions of **L**, can be used to yield more accurate results through a “viscous kernel” (Bro-Nielsen and Gramkow, 1996; Gramkow and Bro-Nielsen, 1997). We assessed all three approaches in this paper.

An Eulerian frame of reference describes the non-linear warp field variables through fixed positions **x** associated with time dependent displacement vectors **u**(**x**, *t*) such that the deformed position at time *t* is described as **x**−**u**(**x**, *t*) (Christensen et al., 1996). The material derivative *d/dt* provides the instantaneous rate of change a point **x** of the grid observes at time *t*. A particle of the viscous body flowing through position **x** at that time will have a velocity **v** described by:

(4) $$v=\frac{du}{dt}=\frac{\partial u}{\partial t}+\sum_{i=1}^{3}v_{i}\frac{\partial u}{\partial x_{i}}\Leftrightarrow \frac{\partial u}{\partial t}=R=v-\sum_{i=1}^{3}v_{i}\frac{\partial u}{\partial x_{i}}$$

where **v** = (*v*_1_, *v*_2_, *v*_3_) and **x** = (*x*_1_, *x*_2_, *x*_3_).

The sum element in Equation 4 accounts for the deformation applied on the body and is zero when the body and the reference grid match. As a consequence, the partial derivative of the deformation **u** with respect to time yields a perturbation field **R** that is used to update the warping. The deformation field **u** is updated for iteration *k* + 1 of the registration algorithm by **R** such that:

(5) $$u^{\left(k+1\right)}=u^{\left(k\right)}+\Delta u^{\left(k\right)}=u^{\left(k\right)}+R^{\left(k\right)}\;\cdot \;\Delta T^{\left(k\right)}$$

where Δ*T*^(*k*)^ is an iteration dependent time step, thus performing an explicit Euler integration of the perturbation vectors, which in themselves form a velocity field. The choice of time step will depend on the maximum value of the perturbation field, ∥**R**^(*k*)^∥_max_. In the current work, Δ*T*^(*k*)^ was chosen such that a maximum displacement value *m* was enforced for each iteration (D'Agostino et al., 2003):

(6) $$\Delta T^{\left(k\right)}=\frac{m}{∥R^{\left(k\right)}{∥}_{\max}}$$

### B. Topology preservation

The determinant of the Jacobian of the deformation field must at all times be positive in order to ensure that topology is preserved (Christensen et al., 1996). This is ensured by regridding the deformation field every time this determinant crosses a certain threshold. Details of how this is done can be found in the supplementary material.

### Viscous fluid assessment

The viscous fluid registration method was used to register gray matter probability maps in the context of VBM analyses of clinical cohorts. The first set of analyses was performed using groups of healthy controls (*n* = 18), Alzheimer's disease (AD) (*n* = 19), semantic dementia (SD) (*n* = 10) and behavioral variant frontotemporal dementia subjects (bvFTD) (*n* = 8). Subjects in each of the disease groups were diagnosed according to standard criteria (McKhann et al., 1984; Neary et al., 1998). These subjects made up Set A. This diverse set of atrophy profiles—AD, SD, and bvFTD—allowed for a thorough assessment of the proposed viscous fluid method in contrast to DARTEL and a standard SPM method. Demographic information about these groups can be found in the supplementary material (**Table S1**). Hippocampal volumes had been measured on the AD and control cohorts of Set A for a previous study (Pengas et al., 2010), and are presented in Table 1.

**Table 1:** **Hippocampal volumes for subjects used in the AD VBM study of Set A, normalized to the mean total intracraneal volume (TIV) of the control cohort**.

	**Right hippocampus volume (mm^3^)**	**Left hippocampus volume (mm^3^)**
Controls	1667 ± 301.0	1574 ± 214.4
AD	1399 ± 294.3 (−16.1%)	1270 ± 369.0 (−19.3%)

*Values presented are mean ± standard deviation. Average percentage volume reduction relative to controls is shown in brackets. Values for the AD subjects are significantly lower than controls (p < 0.05, one-tail t-test). TIV was not statistically different between cohorts (not shown)*.

In order to not confine the assessment to a set of scans sharing the same acquisition parameters, another set of scans (designated Set B) was also used. Set B comprised controls (*n* = 21), AD subjects (*n* = 16), SD subjects (*n* = 10), and *n* = 17 patients diagnosed with mild cognitive impairment (MCI). The demographic details of these groups are listed in the supplementary material (**Table S2**). Hippocampal volumes were measured for all cohorts of Set B and are presented in Table 2. Details of how these volumes were obtained can be found in the supplementary material.

**Table 2:** **Hippocampal volumes for Controls, as well as for AD and MCI subjects in Set B, normalized to the mean TIV of the control cohort**.

	**Right hippocampus volume (mm^3^)**	**Left hippocampus volume (mm^3^)**
Controls	1286 ± 157.5	1206 ± 209.6
AD	986 ± 192.9 (−23.4%)	886 ± 166.2 (−26.6%)
MCI	1037 ± 124.4 (−19.4%)	1008 ± 101.3 (−16.4%)

*Values presented are mean ± standard deviation. Average percentage volume reduction is shown in brackets. Values are significantly lower than controls in all patient cohorts (p < 0.05 one-tail t-test). TIV was not statistically different between cohorts (not shown)*.

Set A was acquired with a 1.5 T GE Signa MRI scanner (GE Medical Systems, Milwaukee, WI). Volumetric *T*_1_-weighted images were coronally acquired using SPGR (pixel dimensions 0.86 × 0.86 mm^2^, slice thickness 1.5 or 1.8 mm). Set B was acquired on a Siemens Trio 3T system (Siemens Medical Systems, Erlangen, Germany) using a 3D MPRAGE pulse sequence for the acquisition of volumetric *T*_1_-weighted images with 144 slices, 192 × 192 matrix dimensions and 1.25 mm^3^ voxel size. Scans were preprocessed according to a previously described pipeline (Acosta-Cabronero et al., 2008) (see supplementary material).

All scans were registered and segmented using the unified segmentation model provided in SPM8 (Ashburner and Friston, 2005). Probability maps of GM, WM, and CSF were also obtained in native space from all subjects for automated total intracranial volume (TIV) estimation (Pengas et al., 2008).

Subjects were also processed with the DARTEL algorithm, designated DARTEL_pre_ when used with preprocessed scans, using default parameters and modulated gray matter probability maps. The output probability maps of the DARTEL algorithm were used in all subsequent analyses without any further processing.

### Viscous fluid registration

Heuristic tests suggested that the eigenfunction-based kernel approximation was faster than SORA, while providing better results than the gaussian kernel approximation. As such, this was the method of choice in this paper. The others methods, however, presented similar results, which will not be discussed herein. All gray matter probability maps, normalized and resliced to an isotropic resolution of 2 mm^3^ were registered with the viscous fluid method using the eigenfunction-based approximation to the linear operator to solve the differential equation. This viscous fluid method will be named “Fluid” from this point on.

For all sets of scans, a fixed random gray matter probability map from the control cohort was used as the target to which all other probability maps were registered. Given that these gray matter probability maps are already registered to a template, no further linear registration was performed.

### VBM analyses

A two-group *t*-test comparison was made between each diseased cohort and the relevant control group (i.e., with the same acquisition parameters). The control target was also included in the control group for the statistical analyses.

A relative threshold mask of 0.2 was used for all studies, except where otherwise stated. Scans were smoothed with an 8 mm full width half maximum (FWHM) Gaussian kernel. All analyses were also performed with 6 and 10 mm FWHM kernels, but as results were very similar to those obtained with the 8 mm kernel these are not shown or discussed. All tests were performed with total intracraneal volume (TIV) as a nuisance covariate.

The analyses of the scans of Set A included both gray matter probability maps from raw (not preprocessed) volumes and preprocessed volumes. Set B was only analysed using preprocessed scans. All probability maps were modulated by the Jacobian determinant of the non-linear viscous transformation. The modulation step was required for consistency with the original probability maps; moreover, the fine deformation fields result in determinant values that contain important information about local volume changes in the brain. All analyses had a statistical threshold of *p*_FWE corrected_ = 0.05, unless the resulting glass brains were blank (or showed only noise), in which case the threshold of *p*_uncorrected_ = 0.001 was used. The extent threshold *k* was set at 0 for all analyses. No correction was made for the differing initial voxel dimensions in Set A as this was a systematic error introduced in all studies and can be discounted when comparing the results (Pereira et al., 2008). Results of DARTEL, DARTEL_pre_, Fluid and Fluid_pre_ were analysed in their own template space.

### Registration assessment

Registration quality was assessed by comparing the results of the viscous fluid re-registration analyses with those of SPM8's unified segmentation and DARTEL.

A quantitative analysis of the registration performance was also performed using a subgroup of Set A that had fiducial points placed on the original scans as part of a previous study (Pereira et al., 2010). A set of 20 locations were chosen for fiducial marker placement. The consistency of location for each fiducial marker cluster was analysed with three metrics–the degree of dispersion error after warping in the direction of greatest location uncertainty (λ_1_), and the extent to which dispersion was distributed along a given plane (R_1_). The first metric (λ_1_) is similar to a standard deviation of the registration error on a specific location—ideally, it should be zero. The second metric (R_1_) is a ratio between the amount of registration misalignment in the main error direction and the total sum of errors in all directions—it complements the amount of error by providing information on the anisotropy of that error, i.e., if the misalignment has a preferential direction (anisotropic) or if it is randomly distributed in space (isotropic). The resulting λ_1_ and R_1_ values of the fiducial clusters from Fluid and Fluid_pre_ were then compared with the corresponding results from SPM, SPM_pre_, DARTEL, and DARTEL_pre_. Further details of this method and results can be found in the supplementary material.

## Results

### VBM results

**Figure 1:**
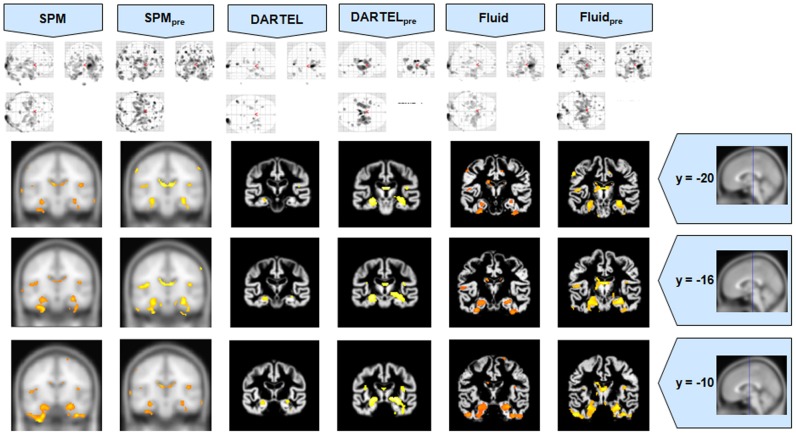
**VBM results for the AD cohort of Set A, with *p*_uncorrected_ = 0.001**. The projection of the results on three coronal slices are presented, at y = −20, −16, and 0 mm, emphasizing the hippocampal region, expected to be atrophic in this pathology and visible in all methods. The entorhinal cortex, however, also likely atrophic, is lost in both DARTEL results. The MNI template was used for SPM and SPM_pre_, and the bespoke target was used for both DARTEL results, as well as for both Fluid results. Due to the use of bespoke template spaces, the presented coronal slices are not strictly comparable across different methods.

**Figure 2:**
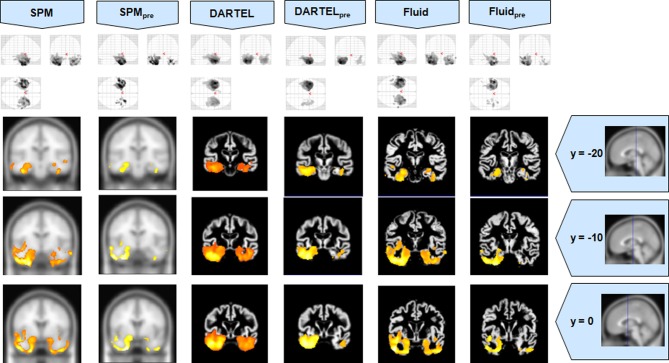
**VBM results for the SD cohort of Set A, with *p*_FWE corrected_ = 0.05**. The projection of the results on three coronal slices are presented, at y = −20, −10, and 0 mm, emphasizing the temporal lobes, expected to be atrophic in this pathology. The lack of anatomical detail in DARTEL is notable, especially around the mesial temporal lobe. This detail is regained with the Fluid methods, with an increase in sensitivity compared to the SPM methods. The MNI template was used for SPM and SPM_pre_, and the bespoke target was used for both DARTEL results, as well as for both Fluid results. Due to the use of bespoke template spaces, the presented coronal slices are not strictly comparable across different methods.

**Figure 3:**
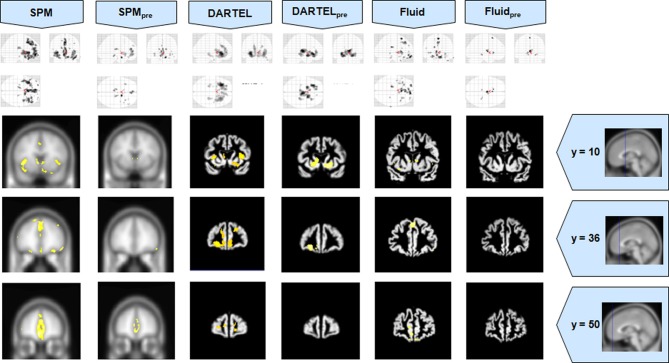
**VBM results for the bvFTD cohort of Set A, with *p*_FWE corrected_ = 0.05**. The projection of the results on three coronal slices are presented, at y = 10, 36, and 50 mm, emphasizing the frontal lobe, expected to be atrophic in this pathology. This was the only case where the use of Fluid methods did not provide tangible benefits; DARTEL was also not more informative than the SPM methods. The MNI template was used for SPM and SPM_pre_, and the bespoke target was used for both DARTEL results, as well as for both Fluid results. Due to the use of bespoke template spaces, the presented coronal slices are not strictly comparable across different methods.

**Figure 4:**
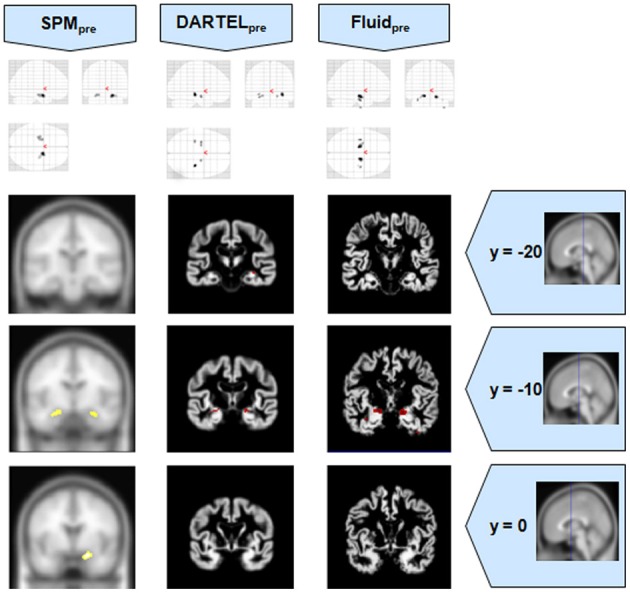
**VBM results for the MCI cohort of Set B, with *p*_FWE corrected_ = 0.05**. The projection of the results on three coronal slices are presented, at y = −20, −10, and 0 mm, emphasizing the hippocampal region, expected to be atrophic in this pathology. Whereas DARTEL detects the atrophy, the Fluid methods are also able to detect it with further anatomical detail. The loss of sensitivity with DARTEL is clear. The MNI template was used for SPM and SPM_pre_, and the bespoke target was used for the DARTEL result, as well as for the Fluid result. Due to the use of bespoke template spaces, the presented coronal slices are not strictly comparable across different methods. A different colour scale was use for Fluid_pre_ to highlight the detected areas.

**Figure 5:**
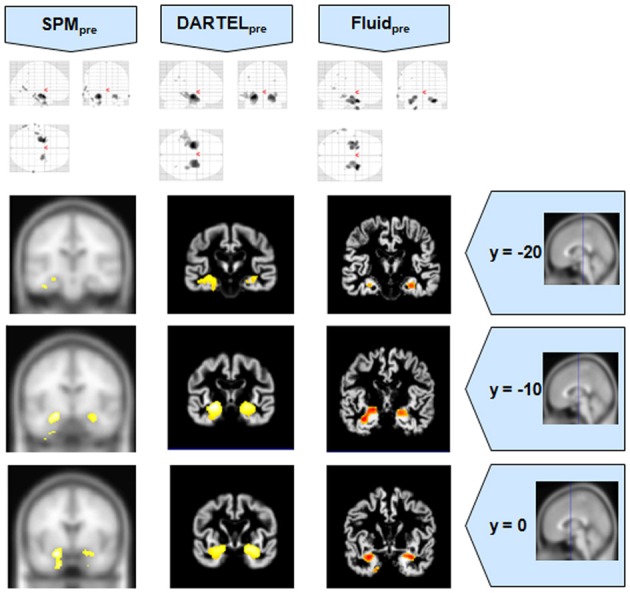
**VBM results for the AD cohort of Set B, with *p*_FWE corrected_ = 0.05**. The projection of the results on three coronal slices are presented, at y = −20, −10, and 0 mm, emphasizing the hippocampal region, expected to be atrophic in this pathology. As before, whereas DARTEL detects the atrophy, the Fluid methods are also able to detect it with further anatomical detail. The MNI template was used for SPM and SPM_pre_, and the bespoke target was used for the DARTEL result, as well as for the Fluid result. Due to the use of bespoke template spaces, the presented coronal slices are not strictly comparable across different methods. A different color scale was use for Fluid_pre_ to highlight the detected areas.

**Figure 6:**
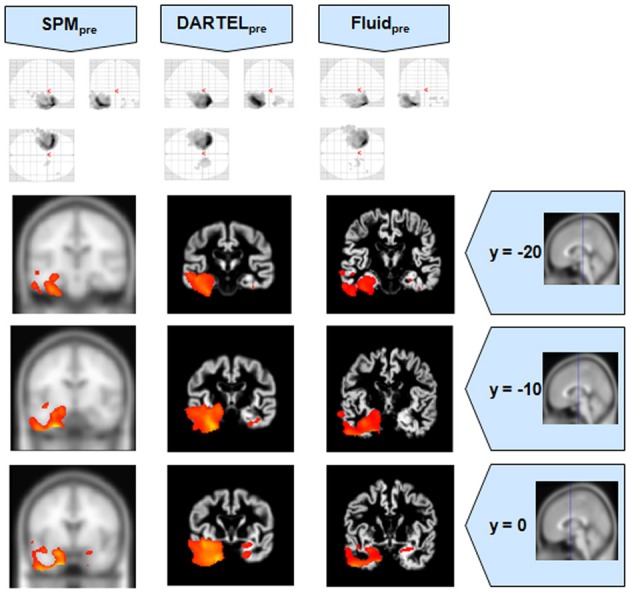
**VBM results for the SD cohort of Set B, with *p*_FWE corrected_ = 0.05**. The projection of the results on three coronal slices are presented, at y = −20, −10, and 0 mm, emphasizing the temporal lobes, expected to be atrophic in this pathology. The gain in sensitivity with the Fluid methods compared to the SPM approaches is clear; the gain in anatomical detail compared to DARTEL is again notable. The MNI template was used for SPM and SPM_pre_, and the bespoke target was used for the DARTEL result, as well as for the Fluid result. Due to the use of bespoke template spaces, the presented coronal slices are not strictly comparable across different methods.

The VBM results for Set A are presented in Figures 1–3, and the results for Set B are in Figures 4–6.

It was observed that VBMs performed with the Fluid registration algorithms showed greater sensitivity than both SPM and DARTEL. This effect was especially evident for mild atrophy scenarios, namely for the AD cohorts. In these cases, hippocampal atrophy—known to be present from the manually measured hippocampi—in Fluid was more extensive than with SPM and (especially) SPM_pre_, without the apparent cost of localization reduction visible in the DARTEL results. In fact, the DARTEL algorithm fared better in terms of eliminating apparent extraneous results in mild atrophy cases, but at the apparent cost of eliminating true positives, a problem previously described in detail (Pereira, 2010) and seen here in Figure 1, where the detection of hippocampal atrophy, in DARTEL without preprocessing, was less pronounced than with the other methods.

Compared to DARTEL, the results obtained with the Fluid algorithms retained greater anatomical detail, comparable to the detail obtained with both SPM methods. DARTEL destroys the anatomical detail of the results, especially in severely atrophic regions that appear as amorphous areas, as seen in the SD analyses shown in Figures 2, 6.

### Assessment of fiducial points

A detailed analysis of the fiducial study has been included in the supplementary material. The λ_1_ values dispersion values for Fluid (both with and without preprocessed scans) remained comparable to those of all other methods for most cases. The Fluid results were very consistent with the other methods. Also as observed previously using SPM and DARTEL (Pereira et al., 2010), there was an interaction between brain pathology and registration difficulties shown across all metrics, with severe focal atrophy still presenting the greatest challenge.

## Discussion

The use of viscous fluid registration algorithms to re-register gray matter probability maps demonstrates potential for VBM analyses. Overall, the VBM results from the analysis of each cohort were consistent with prior knowledge of atrophy profiles. Fluid registration enhanced the sensitivity of VBM while retaining anatomical detail. There was evidence for improvement over both standard SPM and DARTEL in several analyses.

### Registration assessment

The quantitative assessment of Fluid registration when compared to both SPM and DARTEL (further details and results can be found in the supplementary material) showed that all methods were broadly comparable. Despite the good match between target and subject, the registration algorithms seemed to be limited by the inherent variability between subjects that eludes warping. The registration performance values were quite variable across brain regions, and a disease grouping interaction for all methods was visible. The quantitative analysis of fiducial metrics closely resembled the results for SPM5's unified segmentation that were reported previously (Pereira et al., 2010). This is not unexpected, as the viscous fluid registration was based on SPM8's registered probability maps (similar to SPM5's) and the algorithm was also limited to 15 iterations^1^.

Importantly, the observation that the dispersion values of the fiducial markers were not significantly worsened by the Fluid methods suggesting that these registration algorithms are preserving the anatomical validity of the registration—in theory, there is a danger that geometric overfitting could lead to a loss of anatomical validity (i.e., that better fitting comes at the expense of moving anatomical structures). The quantitative results indicate that this did not occur.

### VBM analyses

All methods were able to identify the key abnormalities known from prior knowledge: hippocampal atrophy in AD and MCI groups (Galton et al., 2001; Du et al., 2004; Pennanen et al., 2004; Du et al., 2007); rostral temporal lobe atrophy in SD (Chan et al., 2001; Rosen et al., 2002; Williams et al., 2005; Nestor et al., 2006); and frontal atrophy in bvFTD (Rosen et al., 2002; Williams et al., 2005; Cardenas et al., 2007; Pereira et al., 2009). The preprocessed viscous registration results were, however, more concentrated on these areas of known atrophy in several of the analyses. This might suggest a reduction in false positives though it is important to highlight that, in the absence of ground-truth measurements for unexpected locations, this might also reflect lack of sensitivity to true, albeit unanticipated, abnormalities. On the other hand there was evidence to suggest that adding the viscous registration step offered superior results in detecting true positives and preserving anatomical detail, particularly in contrast to DARTEL. For instance, the AD analysis found fairly restricted hippocampal abnormalities in the temporal lobe using DARTEL. In contrast, the fluid registration method showed blobs extending into the adjacent temporal lobe (Figure 1). This result is far more consistent with previous manual volumetric studies of the entorhinal region (Du et al., 2004; Pennanen et al., 2004) and with knowledge of the spread of neuropathology in very early AD (Braak and Braak, 1991). It should be noted that SPM without DARTEL also detected change in this region. Regarding anatomical precision, the SD analysis also suggested superior performance with viscous registration over DARTEL. As seen in Figures 2, 6, DARTEL identified the rostral temporal abnormality but the blobs were essentially amorphous. In contrast, the viscous method produced results that adhered to the gray matter and were maximal in the rostral inferior surface, again consistent with prior knowledge from manual volumetrics (Chan et al., 2001; Galton et al., 2001; Davies et al., 2004). The difficulties shown by DARTEL when analysing atrophic brains have also been made evident in a recent paper (Ashburner and Friston, 2011), where it is clear that the algorithm underperforms when large deformations are required.

**Figure 7:**
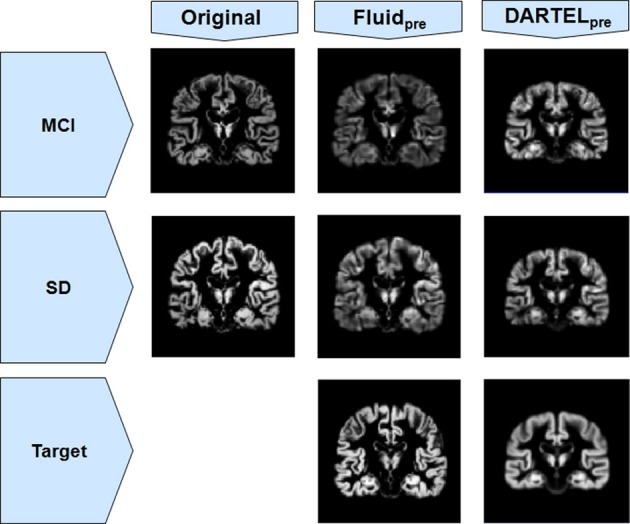
**Gray matter segments before and after registration to a target, for both MCI and SD cohorts from Set B, using both Fluid_pre_ and DARTEL_pre_**. Notice the smoothness of DARTEL_pre_'s target (for the *SD* population only, as an example) compared to the target used in Fluid_pre_.

The loss of anatomical detail due to averaging of subjects with DARTEL is shown in Figure 7. Even though DARTEL iteratively builds the template in order to reduce differences between subjects and to create a sharp average, the effects of blurring were still present. The viscous fluid algorithms, in contrast, generate gray matter probability maps that are locally deformed in order to conform to precise anatomical reference structures, whereas the DARTEL probability map results are degraded, leading to loss of localization power and to VBM results that are smoother and seemingly amorphous (Figures 2, 6). Moreover, DARTEL has more regularization constraints than the viscous registration method presented in this paper that prevent it from creating the very local warps that a viscous fluid method can generate. This creates smooth diffeomorphic deformation fields but leads to limited local warping capabilities.

It must be acknowledged, nevertheless, that the use of a single subject template has some potential disadvantages, namely biasing the registration result for more similar brains. The nature of the gray matter probability maps, however, bypasses some of those limitations as the registered subjects already share a space very similar to the template. To address this concern, the Fluid algorithms were also tested by using as registration target the average of all subjects in each analysis, but results were essentially the same as the ones presented. Even if the templates were similar, the Fluid methods are more lightly regularized and are able to produce fields that can flow more freely than with DARTEL. It must be also noted that the use of a bespoke template in DARTEL, based on the iterative average of the registered gray matter probability maps, is prone to errors if at least one of the probability maps is poorly segmented. Heuristic tests have shown that such an approach may lead to false positives and to the propagation of segmentation errors. This issue will be addressed in future work.

It also important to highlight that while fluid registration yielded results that were more consistent with prior knowledge and/or manual volumetry (AD and MCI) and greater preservation of anatomical detail (SD), the bvFTD analyses did not show a benefit for this technique. All three approaches showed changes in the frontal lobe as would be expected from prior knowledge, though they were least extensive with fluid registration. The frontal lobes are large, and this results suggests that in the face of a large spatial extent of atrophy, the registration step is less important. Interestingly, in this group, it was not DARTEL but rather the default SPM analysis that yielded the most significant and extensive frontal abnormalities.

Finally, in common with many previous clinical VBM studies, this work made use of low numbers of subjects per cohort in order to simulate the real world application as closely as possible. We speculate that low numbers of subjects hinder the quality of a bespoke template—this would explain why DARTEL underperformed so often in the scenarios presented in this article. Moreover, a future application of the work presented in this paper is to make single subject VBM-like analyses possible in a clinical context. Understanding the practical limitations of the available methods when using low numbers of subjects is therefore fundamental to this aim.

## Conclusions

The use of a viscous fluid registration algorithm to re-register the gray matter probability maps produced by the unified segmentation proved to be a useful tool, especially in terms of the qualitative assessment of VBM results. This Fluid registration method was able to provide detailed results with probability maps generated from both unpreprocessed and (especially) preprocessed scans; this was true for both a very focal atrophic cohort such as SD and in milder, more diffuse, atrophy such as AD and MCI. When compared to alternatives, especially DARTEL, which also uses a comparable methodology, the VBM outputs were more contiguous and anatomically localized with the Fluid methods. Additionally, these results suggest that most high-degree of freedom registration algorithm, with very little regularization, may be useful to re-register the probability maps in order to improve VBM results.

### Conflict of interest statement

The authors declare that the research was conducted in the absence of any commercial or financial relationships that could be construed as a potential conflict of interest.

## Footnotes

^1^A more liberal convergence criterion was also tested, up to a maximum of 25 iterations, but the resulting VBM glass brains started to show false positives around the cerebellum. These results suggested that the chosen number of iterations was adequate.
