# Supplementary material for: VBM with viscous fluid registration of gray matter segments in SPM
Source: Front Aging Neurosci. 2013 Jul 15;5:30. doi: 10.3389/fnagi.2013.00030 (PMC3711012; doi:10.3389/fnagi.2013.00030)
Supplement: Supplementary file 1 [file 52497_Pereira_DataSheet1.docx]

**Methods**

*Topology Preservation*

Although the viscous fluid registration method already explicitly regularizes the deformations by the nature of the physical model, it is not guaranteed that the produced warps ensure a homeomorphic transformation. This depends on the maximum displacement value *m* in Eq. 6 (main manuscript) and on the cumulative effect of the total deformation as updated by Eq. 5 (main manuscript), among other factors.

The map of the determinant of the Jacobian must not have a value below a certain positive threshold, usually 0.5 (Christensen et al., 1996). In this work, we have modelled the deformation field differently and, as such, higher values of the determinant of the Jacobian mean compression of the underlying grid, which is the regularisation intends to control. As such, the threshold for regridding was set at a maximum of 1/0.5=2. Whenever one or more voxel locations are detected with values above the threshold, the transformed subject is updated and the deformation and velocity fields are reset to zero across all coordinates, i.e. they are regridded (using cubic interpolation). The Jacobian field is also updated at each regridding step such that **J***_Total_* = (**J**_1_**u**_2_)**J**_2_, where **J**_1_ is the total Jacobian field calculated at the previous regridding step, **u**_2_ is the deformation field produced since the last regridding step, **J**_2_ is the Jacobian of **u**_2_, and **J***_Total_* is the updated Jacobian.

These regridding steps are “lost” iterations of the registration algorithm, i.e. the deformations calculated in the iteration where the determinant of the Jacobian falls below the threshold are not applied to the subject. Moreover, interpolation is required when updating the deformed subject so that the registration algorithm restarts with an already partially registered image. The larger the number of regriddings, the more interpolation errors will be accumulated in the transformed subject, which is not desirable. Finally, for a large enough *m*, the algorithm may fall in a loop where all iterations yield values below the threshold (D'Agostino et al., 2003).

*Preprocessing Method*

1. Skull-stripping was initially performed with the fully automated hybrid watershed algorithm (HWA) (Ségonne et al., 2004) in FreeSurfer v.3.04 (Dale et al., 1999;Fischl et al., 1999), which integrates an atlas-based term constraining the shape of the brain;
2. Stripped volumes are then bias-corrected using the non-parametric non-uniform intensity normalisation v.1.10 (N3) with default arguments (Sled et al., 1998). This algorithm was shown to perform better than SPM5’s bias correction, which is subsequently turned off by imposing the largest possible regularisation on the unified segmentation estimation of the bias field (reference *FWHM* for the bias field set at 150mm and bias regularisation parameter equal to 10);
3. Finally, a fine brain extraction that excludes venous sinuses and cerebrospinal fluid was performed using the brain extraction tool v.2.1 (BET2) (Smith, 2002) in
   FSL v.3.3 (http://www.fmrib.ox.ac.uk/fsl) with vertical gradient *g* set to zero. For Set A, the fractional intensity threshold *f* was optimised for each scan individually using visual inspection of the results. Set B was preprocessed with *f* set to 0.2 as this value was found to be adequate for all subjects under analysis.

*Demographic Information on the Subjects*

| **Characteristics** | **Controls** | **AD** | **SD** | **bvFTD** |
| --- | --- | --- | --- | --- |
| **Age**  (mean ± std) | 63.7 ± 8.75 | 66.6 ± 7.18 | 64.1 ± 7.81 | 58.9 ± 6.10 |
| **Male/Female** (number of subjects) | 9/9 | 6/13 | 6/4 | 6/2 |

Table S1: Demographic information for Set A subjects.

| **Characteristics** | **Controls** | **AD** | **MCI** | **SD** |
| --- | --- | --- | --- | --- |
| **Age**  **(mean ± std)** | 69.7 ± 6.28 | 67.5 ± 6.12 | 71.5 ± 4.88 | 62.2 ± 6.53 |
| **Male/Female (number of subjects)** | 10/11 | 8/8 | 10/7 | 7/3 |

Table S2: Demographic information for the subjects of Set B.

*Hippocampal measurements for Set B*

Using Analyze 8.1, the images were displayed on a Dell 17” 1280 x 1024 TFT LCD screen.  The images were rotated in transverse view where necessary to ensure that the longitudinal fissure was vertical. They were also rotated as necessary in sagittal view to bring the anterior and posterior commissures to the same horizontal level. Starting with the slice just showing the anterior margin of the posterior commissure, the hippocampus on each side was delineated as a region of interest using a tablet drawing device.  The subiculum was not included.  Consecutive slices were similarly delineated until the anterior limit of the hippocampus was reached. In those cases where the head of the hippocampus was hard to distinguish from the amygdala, the image was checked in sagittal view. The total volumes enclosed by the hippocampal slices were derived using the Analyze program. A random selection of subjects was re-measured for intra-rater measurements. Not all intra-rater correspondences were acceptable due to the novelty of the task to the rater. The rater therefore measured them all again, and again a random selection was re-drawn for checking. In addition, those whose measures differed from the first attempt by more than 10% were re-measured as a check. A total of 38 (out of 72) measures were checked. The mean of check measures/final measurements (left and right) was 1.02 (standard deviation right = 0.1, left = 0.09) and the range was 0.85 – 1.2.

*Interpolation Methods*

Trilinear interpolation was used when applying the deformation field to the images. The use of this method of interpolation was justified by observing that the intensity profile of the registered scans was more stable when compared to the original histograms than when using cubic splines. This stability is fundamental when registering grey matter segments, as each voxel contains a tissue belonging probabilistic measure. Cubic interpolation was used only to update the deformation fields, and to translate the higher hierarchical level deformations to the original grid.

*Quantitative Registration Assessment*

This analysis follows the method described in the literature (Pereira et al., 2010), using the exact same subjects. The only difference was that the target was chosen from within each cohort and therefore only nine subjects per cohort were used for the analysis (the target subject was removed in order to avoid a result bias because the target does not undergo viscous fluid registration and, being the reference, the inclusion of its fiducial points may lead to optimistic estimates of performance). The resulting λ_1_ and R_1_ values were then compared with the corresponding results from SPM, SPMpre, DARTEL, and DARTELpre. Comparisons were also made with results from the manual registration for λ_1_ and R_1_ values.

**Results**.

### *Registration Assessment Results*

The results for R_1_ and λ_1_ values can be found in Figures S1, S2, and S3:


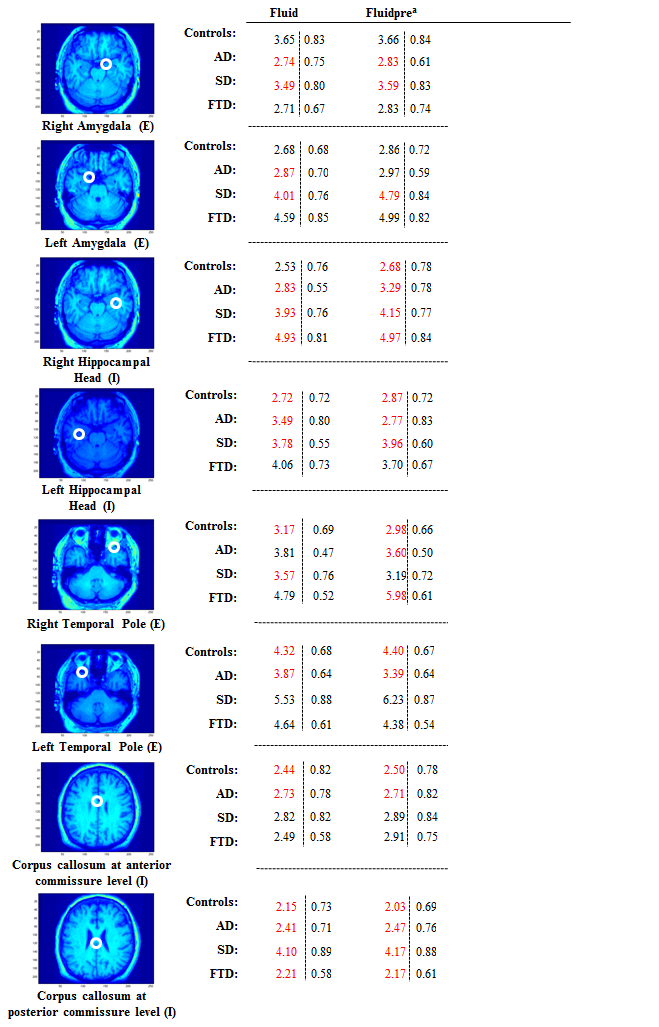


Figure S1: The first column shows the λ_1_ values for manual registration in mm. The other columns show R_1_ | λ_1_ values for all diseased cohorts, after Fluid registration with the raw scans, and after Fluid registration using the unified segmentation approach on the optimally preprocessed scans. External landmarks are marked with (E), internal with (I). Values in red indicate worse dispersion after registration.

^a^ Only eight subjects were used due to failure of the skull stripping algorithm in one.


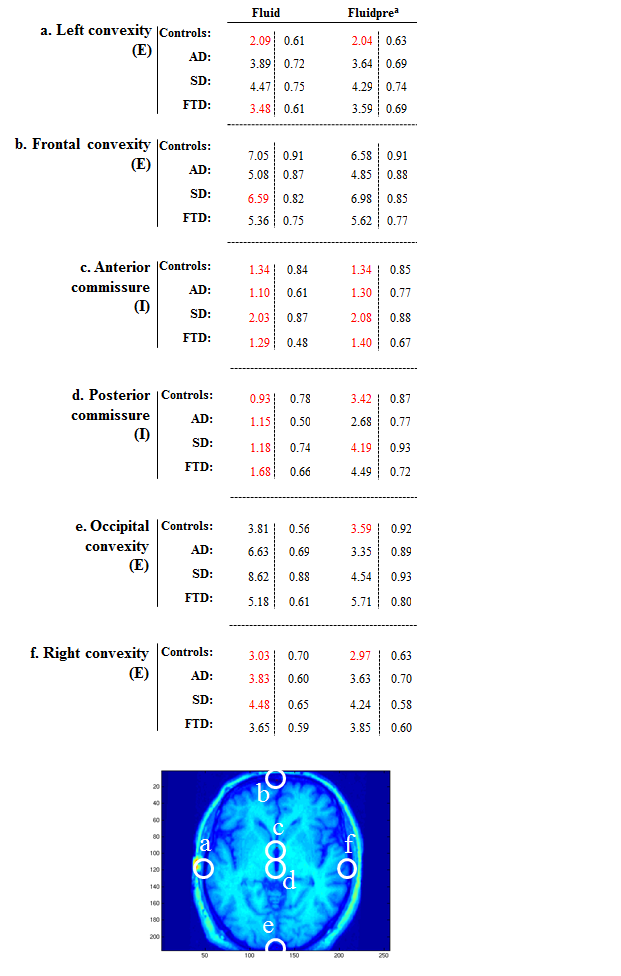


Figure S2: The first column shows the λ_1_ values for manual registration in mm. The other columns show R_1_ | λ_1_ values for all diseased cohorts, after Fluid registration with the raw scans, and after Fluid registration using the unified segmentation approach on the optimally preprocessed scans. External landmarks are marked with (E), internal with (I). Values in red indicate worse dispersion after registration.

^a^ Only eight subjects were used due to failure of the skull stripping algorithm in one.


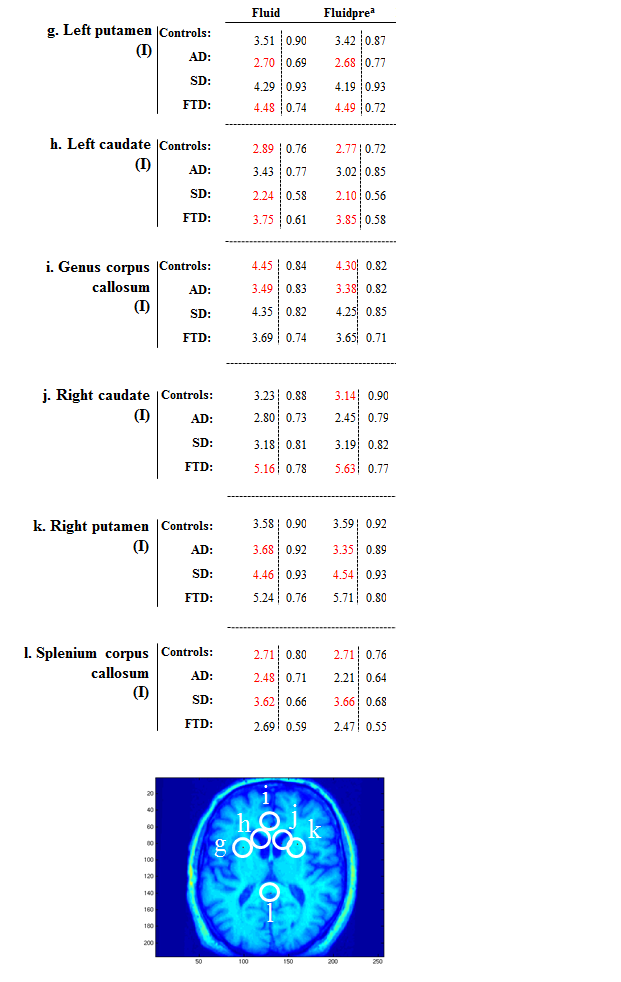


Figure S3: The first column shows the λ_1_ values for manual registration in mm. The other columns show R_1_ | λ_1_ values for all diseased cohorts, after Fluid registration with the raw scans, and after Fluid registration using the unified segmentation approach on the optimally preprocessed scans. All landmarks are internal here (I). Values in red indicate worse dispersion after registration.

^a^ Only eight subjects were used due to failure of the skull stripping algorithm in one.

*Between Cohorts and Within Methods*

Comparing the R_1_ and λ_1_ values using two-tails t-tests, with significance for
p-value < 0.05, the results from Fluid and Fluid_pre_ are remarkably consistent, with no differences detected between cohorts registered with either method. Analysing the differences between cohorts within each method, the results were once again very similar. There was a difference in Fluid_pre_ dispersion values for SD subjects that suggested that λ_1_ is higher than for controls and AD.

*Between Methods and Within Cohorts*

When comparing R_1_ and λ_1_ with other methods, including manual registration for λ_1_, there were no significant differences to report, apart from lower R_1_ values in Fluid when compared to DARTEL and DARTEL_pre_.

*Internal and External Fiducials*

The division of fiducials between internal and external landmarks revealed few other differences. For both Fluid methods, λ_1_ was higher in the external landmarks for AD and SD.

Comparing across methods, the only differences occurred in internal R_1_ values for FTD subjects on both methods, which were lower than the corresponding values in DARTEL. Differences between cohorts were observed with higher external R_1_ values in SD than in AD and FTD in both methods. In Fluid, internal R_1_ was higher in Controls than in AD, and higher in SD than in FTD. In Fluid_pre_, differences were detected for R_1_ and λ_1_ between FTD and AD, having the former lower R_1_ and higher λ_1_.

*Direction of Dispersion*

The visualization of the main eigenvectors can be seen in Figure S4 for the Fluid method. This analysis once again revealed a clear consistency of direction across cohorts, as well as for both Fluid and Fluid_pre_. The directions of variability showed a connection with the structures with which they are associated.


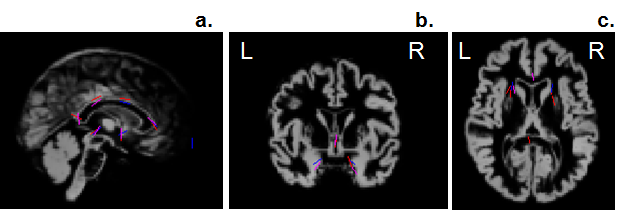


Figure S4: Principal component eigenvectors (×5 for visualization purposes) of the registration error of the Fluid method for all cohorts, projected onto the grey matter segment of a control scan: (**a.**) is a sagittal slice in the mid-plane, (**b.**) is a coronal slice through the amygdala, and (**c.**) is an axial slice through the striatum.

**Discussion**

### *A. Registration Assessment*

The visual assessment is not reflected on the λ_1_ values, as these remained comparable to those of all other methods for most cases. This suggests that, despite the registration strategies implemented, a lower bound is being reached in terms of anatomical alignment. Despite the good match between target and subject, the registration algorithms seem to be limited by the inherent variability between subjects that eludes warping – visual alignment is achieved by severely distorting some anatomical regions.

The proposed methods are nonetheless very consistent with the other assessed methods, suggesting a stable performance. Minimal differences were detected when analysing internal and external landmarks separately. The performance values were nonetheless still quite variable across brain regions, and a disease grouping interacting for all methods was visible. The values obtained with the quantitative analysis closely resembled those of SPM5’s unified segmentation analysed in the previous work (Pereira et al., 2010). This is somewhat expected, as the viscous fluid registration was based on SPM8’s registered segments (similar to SPM5) and the algorithm was also limited at 15 iterations^[[1]](#footnote-1)^. A similar performance in terms of direction of variability is also observed, suggesting that the proposed algorithm is unable to counteract this effect.

**References**

Christensen, G.E., Rabbitt, R.D., and Miller, M.I. (1996). Deformable Templates Using Large Deformation Kinematics. *IEEE Transactions on Image Processing* 5**,** 1435-1447.

D'agostino, E., Maes, F., Vandermeulen, D., and Suetens, P. (2003). A Viscous Fluid Model for Multimodal Non-Rigid Image Registration Using Mutual Information. *Medical Image Analysis* 7**,** 565-575.

Dale, A.M., Fischl, B., and Sereno, M.I. (1999). Cortical Surface-Based Segmentation - I. Segmentation and Surface Reconstruction. *Neuroimage* 9**,** 179-194.

Fischl, B., Sereno, M.I., and Dale, A.M. (1999). Cortical Surface-Based Segmentation - II. Inflation, Flattening, and a Surface-Based Coordinate System. *Neuroimage* 9**,** 195-207.

Pereira, J.M.S., Xiong, L., Acosta-Cabronero, J., Pengas, G., Williams, G.B., and Nestor, P.J. (2010). Registration accuracy for VBM studies varies according to region and degenerative disease grouping. *Neuroimage* 49**,** 2205–2215.

Ségonne, F., Dale, A.M., Busa, E., Glessner, M., Salat, D., Hahn, H.K., and Fischl, B. (2004). A hybrid approach to the skull stripping problem in MRI. *Neuroimage* 22**,** 1060-1075.

Sled, J.G., Zijdenbos, A.P., and Evans, A.C. (1998). A nonparametric method for automatic correction of intensity non-uniformity in MRI data. *IEEE Transactions on Medical Imaging* 17**,** 87-97.

Smith, S.W. (2002). Fast Robust Automated Brain Extraction. *Human Brain Mapping* 17**,** 143-155.

1. A more liberal convergence criterium was also tested, up to a maximum of 25 iterations, but the resulting VBM glass brains started showing a number of false positives. These results suggested that the chosen number of iterations was adequate. [↑](#footnote-ref-1)
